# Melatonin Inhibits Transforming Growth Factor-β1-Induced Epithelial–Mesenchymal Transition in AML12 Hepatocytes

**DOI:** 10.3390/biology8040084

**Published:** 2019-11-11

**Authors:** Jung-Yeon Kim, Jae-Hyung Park, Kiryeong Kim, Jaechan Leem, Kwan-Kyu Park

**Affiliations:** 1 Department of Immunology, School of Medicine, Catholic University of Daegu, Daegu 42472, Korea; jy1118@cu.ac.kr; 2 Department of Physiology, School of Medicine, Keimyung University, Daegu 42601, Korea; physiopark@kmu.ac.kr (J.-H.P.); kileyong93@hanmail.net (K.K.); 3 Department of Pathology, School of Medicine, Catholic University of Daegu, Daegu 42472, Korea; kkpark@cu.ac.kr

**Keywords:** melatonin, transforming growth factor-β1, liver fibrosis, epithelial-mesenchymal transition, reactive oxygen species

## Abstract

Recent studies showed that melatonin, a well-known pineal hormone that modulates the circadian rhythm, exerts beneficial effects against liver fibrosis. However, mechanisms for its protective action against the fibrotic processes remain incompletely understood. Here, we aimed to explore the effects of the hormone on transforming growth factor-β1 (TGF-β1)-stimulated epithelial–mesenchymal transition (EMT) in AML12 hepatocytes. Pretreatment with melatonin dose-dependently reversed downregulation of an epithelial marker and upregulation of mesenchymal markers after TGF-β1 stimulation. Additionally, melatonin dose-dependently suppressed an increased phosphorylation of Smad2/3 after TGF-β1 treatment. Besides the canonical Smad signaling pathway, an increase in phosphorylation of extracellular signal-regulated kinase 1/2 and p38 was also dose-dependently attenuated by melatonin. The suppressive effect of the hormone on EMT stimulated by TGF-β1 was not affected by luzindole, an antagonist of melatonin membrane receptors, suggesting that its membrane receptors are not required for the inhibitory action of melatonin. Moreover, melatonin suppressed elevation of intracellular reactive oxygen species (ROS) levels in TGF-β1-treated cells. Finally, TGF-β1-stimulated EMT was also inhibited by the antioxidant N-acetylcysteine. Collectively, these results suggest that melatonin prevents TGF-β1-stimulated EMT through suppression of Smad and mitogen-activated protein kinase signaling cascades by deactivating ROS-dependent mechanisms in a membrane receptor-independent manner.

## 1. Introduction

Liver fibrosis is attributed to aberrant deposition of extracellular matrix (ECM) in liver parenchyma that leads to hepatic dysfunction. Accumulating evidence suggests that multiple cellular and molecular pathways are implicated in the pathophysiology of liver fibrosis [1]. Among them, epithelial–mesenchymal transition (EMT) of hepatocytes to myofibroblasts is suggested as a crucial process in liver fibrosis [2,3,4,5], although there remains some controversy [6,7]. Therefore, blocking EMT of hepatocytes might serve as a potential therapeutic or preventive approach against liver fibrosis.

The pineal hormone melatonin is well known to modulate the circadian rhythm. Furthermore, the hormone has been shown to display beneficial effects including anti-oxidant and anti-inflammatory properties in various diseases [8]. Thus, increasing attention has been paid to the hormone’s potential as a nutraceutical for prevention and treatment of human diseases [9,10]. Emerging evidence suggests that melatonin also exhibits anti-fibrotic effects in various tissues [11]. Interestingly, melatonin was found to exert protective effects on liver fibrosis induced by carbon tetrachloride (CCl4) [12,13,14], thioacetamide [15], or high-fat diet [16]. Melatonin also ameliorated the hepatic fibrotic processes in a bile duct ligation-induced model [17] and a genetic model of primary sclerosing cholangitis [18]. However, cellular and molecular mechanisms for its anti-fibrotic effect on liver fibrosis remain incompletely understood.

In this study, we aimed to examine the effect of melatonin on transforming growth factor-β1 (TGF-β1)-stimulated EMT of hepatocytes and explored its underlying mechanisms.

## 2. Materials and Methods

### 2.1. Cell Culture and Cell Treatments

AML12 cells, a mouse hepatocyte cell line, were purchased from the American Type Culture Collection (Rockville, MD, USA) and maintained in DMEM/F-12 medium supplemented with 10% fetal bovine serum, insulin-transferrin-selenium and dexamethasone at 37 °C under 5% CO₂ and 95% air. To examine the effects of melatonin (Sigma-Aldrich, St. Louis, MO, USA) on EMT stimulated by TGF-β1, cells were preincubated with melatonin (0.1 mM or 1 mM) or 0.1% dimethyl sulfoxide (DMSO; vehicle) for 30 min and then treated with TGF-β1 (2 ng/mL; R&D Systems, Minneapolis, MN, USA) for 24 or 48 h. In another experiment, cells were preincubated with 1 mM melatonin or 0.6% dimethyl sulfoxide (DMSO; vehicle) for 30 min in the presence or absence of 100 μM luzindole (Sigma-Aldrich) and then treated with TGF-β1 (2 ng/mL) for 48 h. In addition, to evaluate the effects of N-acetylcysteine (NAC; Sigma-Aldrich), cells were pretreated with 10 mM NAC for 30 min and then treated with TGF-β1 (2 ng/mL) for 48 h.

### 2.2. Western Blot Analysis

Western blotting was carried out according to the method described previously [19]. In brief, protein samples were separated by sodium dodecyl sulfate polyacrylamide gel electrophoresis (SDS-PAGE) and then transferred from the gels onto nitrocellulose membranes (Millipore, Billerica, MA, USA). The membranes were incubated with a primary antibody overnight at 4 °C. After washing, the membranes were probed with secondary antibodies conjugated to a horseradish peroxidase for 1 h at room temperature. The primary antibodies used in this study were as follows: anti-E-cadherin (1:1000; 610182; BD Biosciences, San Jose, CA, USA), anti-collagen I (1:1000; ab34710; Abcam, Cambridge, MA, USA), anti-fibronectin (1:1000; ab2413; Abcam), anti-α-smooth muscle actin (α-SMA; 1:1000; A2547; Sigma-Aldrich), anti-vimentin (1:1000; ab92547, Abcam), anti-Smad2/3 (1:1000; #3102; Cell Signaling, Danvers, MA, USA), anti-p-Smad2/3 (1:1000; #8828; Cell Signaling), anti-extracellular signal-regulated kinase 1/2 (ERK1/2; 1:1000; #9102; Cell Signaling), anti-p-ERK1/2 (1:1000; #4370; Cell Signaling), anti-c-Jun N-terminal kinase 1/2 (JNK1/2; 1:1000; #9252; Cell Signaling), anti-p-JNK1/2 (1:1000; #9251; Cell Signaling), anti-p38 (1:1000; #8690; Cell Signaling), anti-p-p38 (1:1000; #9215; Cell Signaling), and anti-β-actin (1:3000; A2228; Sigma-Aldrich) antibody. The signals were detected with enhanced chemiluminescence reagents and analyzed using an image analyzer. The protein expression level was normalized against β-actin.

### 2.3. Quantitative Real-Time Reverse Transcription Polymerase Chain Reaction (RT-PCR)

Total RNA extracted with the TRIzol reagent (Thermo Fisher Scientific, Waltham, MA, USA) was reverse-transcribed by using the AccuPower RT Premix (Bioneer, Daejeon, Korea) and oligo (dT) 18 primers. Quantitative real-time RT-PCR was carried out using the Real-Time PCR 7500 system (Applied Biosystems, Foster city, CA, USA) with Power SYBR Green PCR Master Mix (Applied Biosystems). The primers used are listed in Table 1. Glyceraldehyde 3-phosphate dehydrogenase (GAPDH) was used as the reference gene.

### 2.4. Evaluation of Intracellular ROS

Intracellular ROS levels were assessed using the 2′,7′-dichlorodihydrofluorescein diacetate (DCFDA)- Cellular ROS Assay Kit (Abcam) according to the manufacturer’s instructions.

### 2.5. Statistical Analysis

Data are represented as the mean ± standard error of the mean (SEM). Statistical significance was analyzed using one-way ANOVA followed by Bonferroni’s post-hoc tests to evaluate the differences between experimental groups. *p* ˂ 0.05 was considered to be statistically significant.

## 3. Results

### 3.1. Melatonin Prevents TGF-β1-Stimulated EMT in AML12 Hepatocytes

**Figure 1:**
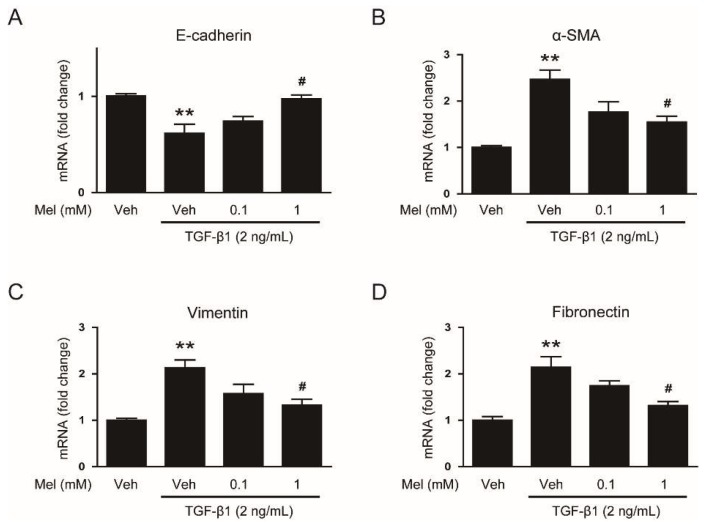
Effects of melatonin on mRNA expression of epithelial–mesenchymal transition (EMT) markers in transforming growth factor-β1 (TGF-β1)-treated hepatocytes. AML12 hepatocytes were preincubated with melatonin (Mel; 0.1 mM or 1 mM) or vehicle (Veh; 0.1% dimethyl sulfoxide) for 30 min and then treated with TGF-β1 (2 ng/mL) for 48 h. Relative mRNA levels of E-cadherin (**A**), α-smooth muscle actin (α-SMA) (**B**), vimentin (**C**), and fibronectin (**D**). All data are presented as the mean ± standard error of the mean (SEM). ** *p* < 0.01 vs. vehicle-treated cells (Veh). ^#^ *p* < 0.05 vs. cells treated with TGF-β1 alone.

**Figure 2:**
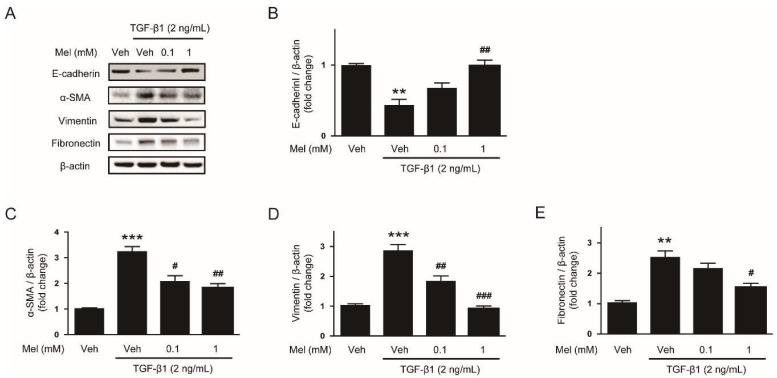
Effects of melatonin on protein levels of EMT markers in TGF-β1-treated hepatocytes. AML12 hepatocytes were preincubated with melatonin (Mel; 0.1 mM or 1 mM) or vehicle (Veh; 0.1% dimethyl sulfoxide) for 30 min and then treated with TGF-β1 (2 ng/mL) for 48 h. (**A**) Western blot image of the expression of E-cadherin, α-SMA, vimentin, fibronectin, and β-actin. The graphs show densitometric quantification of E-cadherin (**B**), α-SMA (**C**), vimentin (**D**), and fibronectin (**E**) normalized against β-actin. All data are presented as the mean ± SEM. ** *p* < 0.01 and *** *p* < 0.001 vs. vehicle-treated cells (Veh). ^#^ *p* < 0.05, ^##^ *p* < 0.01, and ^##^^#^ *p* < 0.001 vs. cells treated with TGF-β1 alone.

To explore the effects of melatonin on EMT stimulated by TGF-β1, we first examined mRNA levels of EMT markers in AML12 cells pretreated with or without melatonin (0.1 mM or 1 mM) after TGF-β1 stimulation. Cells treated with TGF-β1 alone exhibited a reduction in mRNA level of E-cadherin (Figure 1A), a prototypical epithelial cell marker, and an elevation in levels of mesenchymal markers, including α-SMA (Figure 1B), vimentin (Figure 1C) and fibronectin (Figure 1D). These findings indicate that AML12 cells lose their epithelial features and obtain mesenchymal phenotype by TGF-β1. Interestingly, these effects of TGF-β1 were dose-dependently reversed by pretreatment with melatonin (Figure 1A–D). Western blotting confirmed that increased protein levels of the EMT markers after TGF-β1 stimulation were also dose-dependently reversed by melatonin (Figure 2A–E). Collectively, these findings suggest that the hormone significantly inhibits EMT stimulated by TGF-β1 in AML 12 hepatocytes.

### 3.2. Melatonin Attenuates TGF-β1-Stimulated Smad and MAPK Signaling Pathways

**Figure 3:**
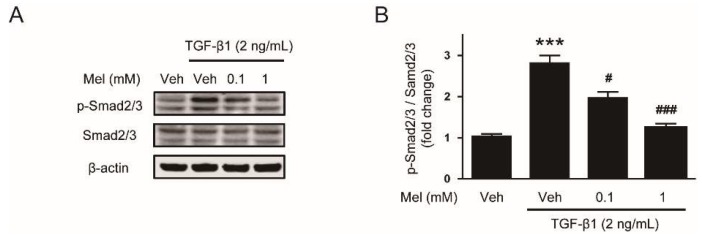
Effects of melatonin on the Smad signaling pathway in TGF-β1-treated hepatocytes. AML12 hepatocytes were preincubated with melatonin (Mel; 0.1 mM or 1 mM) or vehicle (Veh; 0.1% dimethyl sulfoxide) for 30 min and then treated with TGF-β1 (2 ng/mL) for 24 h. (**A**) Western blot image of the expression of p-Smad2/3, Smad2/3, and β-actin. (**B**) Densitometric quantification of p-Smad2/3 normalized against Smad2/3. All data are presented as the mean ± SEM. *** *p* < 0.001 vs. vehicle-treated cells (Veh). ^#^ *p* < 0.05 and ^##^^#^ *p* < 0.001 vs. cells treated with TGF-β1 alone.

**Figure 4:**
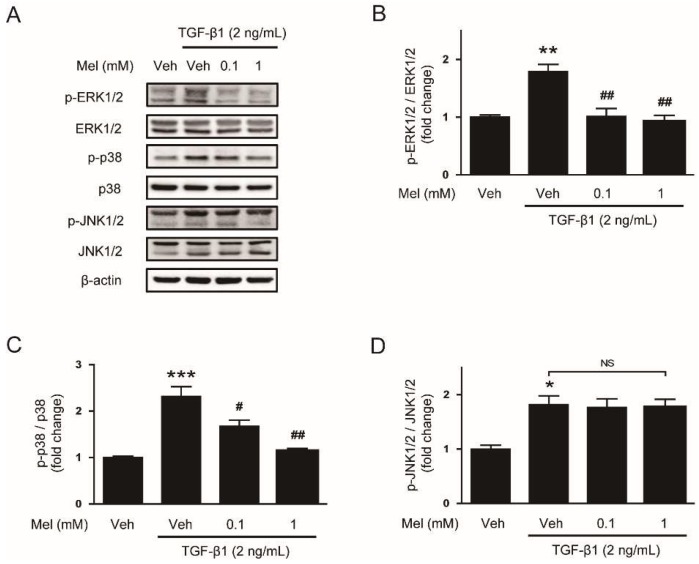
Effects of melatonin on the mitogen-activated protein kinase (MAPK) signaling pathway in TGF-β1-treated hepatocytes. AML12 hepatocytes were preincubated with melatonin (Mel; 0.1 mM or 1 mM) or vehicle (Veh; 0.1% dimethyl sulfoxide) for 30 min and then treated with TGF-β1 (2 ng/mL) for 24 h. (**A**) Western blot image of the expression of p-extracellular signal-regulated kinase 1/2 (p-ERK1/2), ERK1/2, p-p38, p38, p-c-Jun N-terminal kinase 1/2 (p-JNK1/2), JNK1/2, and β-actin. The graphs show densitometric quantification of p-ERK1/2 (**B**), p-p38 (**C**), and p-JNK1/2 (**D**) normalized against the total level of each protein. All data are presented as the mean ± SEM. * *p* < 0.05, ** *p* < 0.05, and *** *p* < 0.001 vs. vehicle-treated cells (Veh). ^#^ *p* < 0.05 and ^##^ *p* < 0.01 vs. cells treated with TGF-β1 alone. NS: not significant.

To investigate mechanisms for the suppressive effect of melatonin on EMT stimulated by TGF-β1, we next evaluated the effects of melatonin on TGF-β1-stimulated Smad signaling. Interaction of TGF-β1 with its receptor on the cell membrane results in Smad2/3 phosphorylation [20]. The phosphorylated Smad proteins interact with Smad4 and then transport into the nucleus where the complex can increase transcription of fibrosis-related genes. We found that preincubation with melatonin dose-dependently inhibited Smad2/3 phosphorylation after TGF-β1 treatment (Figure 3A,B). Besides the canonical Smad signaling cascade, TGF-β1 also activates non-Smad signaling cascades such as MAPK signaling pathways [21]. We found that increased phosphorylation of ERK1/2 and p38 after TGF-β1 stimulation was also dose-dependently attenuated by melatonin, whereas JNK1/2 phosphorylation was not affected (Figure 4A–D). Collectively, these results suggest that melatonin significantly inhibits TGF- β1-stimulated Smad and MAPK signaling cascades.

### 3.3. Suppressive Effect of Melatonin on EMT Stimulated by TGF-β1 Is Independent of Its Membrane Receptors

**Figure 5:**
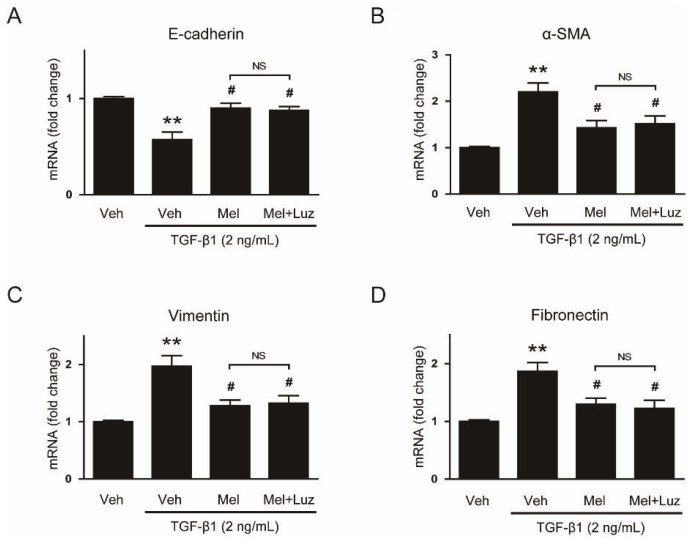
Effects of luzindole on mRNA expression of EMT markers in TGF-β1-treated hepatocytes preincubated with melatonin. AML12 hepatocytes were preincubated with melatonin (Mel; 1 mM) or vehicle (Veh; 0.6% dimethyl sulfoxide) for 30 min in the presence or absence of luzindole (Luz; 100 μM) and then treated with TGF-β1 (2 ng/mL) for 48 h. Relative mRNA levels of E-cadherin (**A**), α-SMA (**B**), vimentin (**C**), and fibronectin (**D**). All data are presented as the mean ± SEM. ** *p* < 0.01 vs. vehicle-treated cells (Veh). ^#^ *p* < 0.05 vs. cells treated with TGF-β1 alone. NS: not significant.

**Figure 6:**
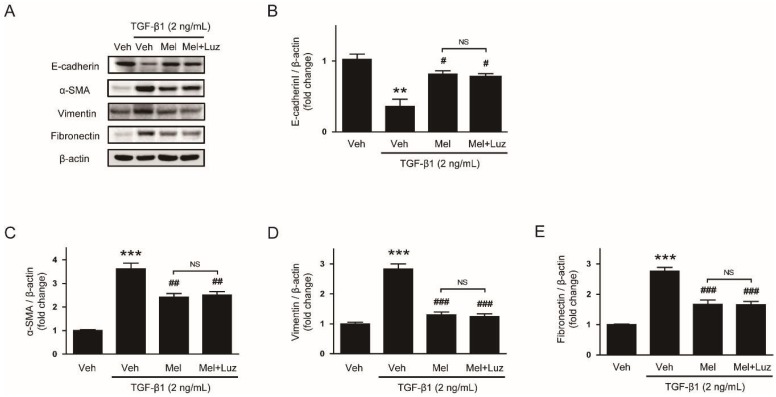
Effects of luzindole on protein levels of EMT markers in TGF-β1-treated hepatocytes preincubated with melatonin. AML12 hepatocytes were preincubated with melatonin (Mel; 1 mM) or vehicle (Veh; 0.6% dimethyl sulfoxide) for 30 min in the presence or absence of luzindole (Luz; 100 μM) and then treated with TGF-β1 (2 ng/mL) for 48 h. (**A**) Western blot image of the expression of E-cadherin, α-SMA, vimentin, fibronectin, and β-actin. The graphs show densitometric quantification of E-cadherin (**B**), α-SMA (**C**), vimentin (**D**), and fibronectin (**E**) normalized against β-actin. All data are presented as the mean ± SEM. ** *p* < 0.01 and *** *p* < 0.001 vs. vehicle-treated cells (Veh). ^#^ *p* < 0.05, ^##^ *p* < 0.01, and ^##^^#^ *p* < 0.001 vs. cells treated with TGF-β1 alone. NS: not significant.

Melatonin exhibits various hormonal effects through both membrane receptor-dependent and independent mechanisms [8]. To investigate whether its membrane receptors are required for the inhibitory action of melatonin on EMT stimulated by TGF-β1, we examined the effects of luzindole, a known antagonist of melatonin membrane receptors, at a routinely used concentration (100 μM) [22,23], on the action of melatonin (1mM). We found that the effects of the hormone on mRNA levels of EMT markers after TGF-β1 stimulation were not affected by luzindole (Figure 5A–D). Western blotting confirmed that luzindole did not affect protein expressoin of the EMT markers (Figure 6A–E). Collectively, these results suggest that melatonin receptors are not required for the suppressive action of melatonin on EMT stimulated by TGF-β1 in AML12 hepatocytes.

### 3.4. Suppressive Effect of Melatonin on EMT Stimulated by TGF-β1 Is Mediated by Suppressing ROS-Dependent Mechanisms

**Figure 7:**
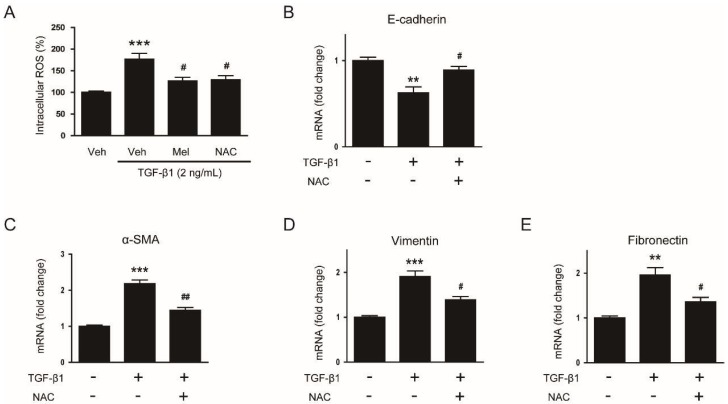
Effects of N-acetylcysteine (NAC) on reactive oxygen species (ROS) generation and mRNA expression of EMT markers in TGF-β1-treated hepatocytes. (**A**) AML12 hepatocytes were preincubated with melatonin (Mel; 1mM), NAC (10 mM), or vehicle (Veh; 0.1% dimethyl sulfoxide) for 30 min and then treated with TGF-β1 (2 ng/mL) for 48 h. Intracellular ROS was measured using the 2′,7′-dichlorodihydrofluorescein diacetate assay. *** *p* < 0.001 vs. vehicle-treated cells (Veh). ^#^ *p* < 0.05 vs. cells treated with TGF-β1 alone. (**B**–**E**) AML12 hepatocytes were preincubated with NAC (10 mM) for 30 min and then treated with 2 ng/mL TGF-β1 for 48 h. Relative mRNA levels of E-cadherin (**B**), α-SMA (**C**), vimentin (**D**), and fibronectin (**E**). All data are presented as the mean ± SEM. ** *p* < 0.01 and *** *p* < 0.001 vs. non-treated cells. ^#^ *p* < 0.05 and ^##^ *p* < 0.01 vs. cells treated with TGF-β1 alone.

The antioxidant activity of melatonin has been critically implicated in its membrane receptor-independent mechanisms [8]. To investigate whether suppressive action of melatonin on TGF-β1-stimulated EMT is mediated by suppressing ROS-dependent mechanisms, we first examined the effect of melatonin and NAC, a well-known antioxidant, on TGF-β1-stimulated ROS generation in AML12 cells. Pretreatment with melatonin or NAC largely suppressed the increased intracellular level of ROS in TGF-β1-treated cells (Figure 7A), indicating that these molecules effectively reduce ROS generation in TGF-β1-treated mouse hepatocytes.

**Figure 8:**
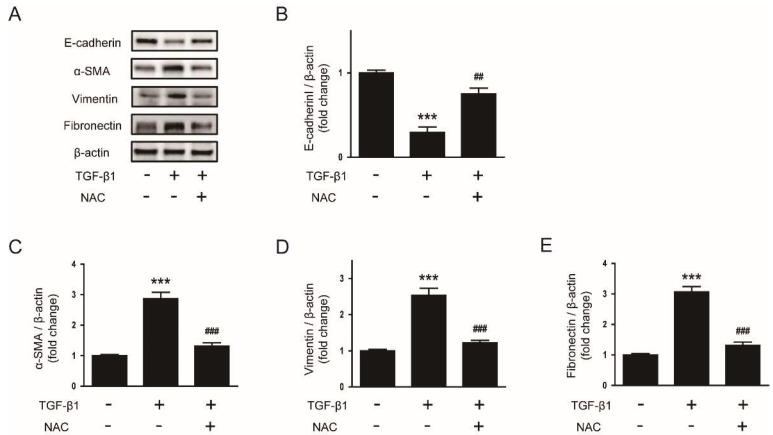
Effects of NAC on protein levels of EMT markers in TGF-β1-treated hepatocytes. AML12 hepatocytes were preincubated with NAC (10 mM) for 30 min and then treated with TGF-β1 (2 ng/mL) for 48 h. (**A**) Western blot image of the expression of E-cadherin, α-SMA, vimentin, fibronectin, and β-actin. The graphs show densitometric quantification of E-cadherin (**B**), α-SMA (**C**), vimentin (**D**), and fibronectin (**E**) normalized against β-actin. All data are presented as the mean ± SEM. *** *p* < 0.001 vs. non-treated cells. ^#^^#^ *p* < 0.01 and ^##^^#^ *p* < 0.001 vs. cells treated with TGF-β1 alone.

We next evaluated whether the antioxidant NAC can also prevent EMT stimulated by TGF-β1 in AML12 cells. Pretreatment with NAC significantly reversed the changes in mRNA levels of E-cadherin (Figure 7B), α-SMA (Figure 7C), vimentin (Figure 7D) and fibronectin (Figure 7E) after TGF-β1 stimulation. Western blotting confirmed that protein levels of the EMT markers were also significantly reversed by NAC (Figure 8A–E). Collectively, these findings suggest that the compound significantly inhibits EMT stimulated by TGF-β1 in AML 12 hepatocytes.

## 4. Discussion

In this study, we showed that melatonin inhibits EMT stimulated by TGF-β1 in AML12 hepatocytes. Pretreatment with melatonin dose-dependently reversed TGF-β1-induced changes in expression of EMT markers. These effects of melatonin were associated with inhibition of both Smad and MAPK signaling cascades. Moreover, we observed that treatment with an antagonist of melatonin membrane receptors failed to block the effects of melatonin, indicating that melatonin receptors are dispensable for the inhibitory action of melatonin on EMT stimulated by TGF-β1. Furthermore, we provided evidence that the inhibitory action of melatonin on EMT stimulated by TGF-β1 is presumably mediated by suppressing ROS-dependent mechanisms.

Melatonin is a well-known pituitary hormone that modulates the circadian rhythm. Furthermore, accumulating evidence suggests that the hormone exerts protective effects against various diseases mainly through its anti-oxidant and anti-inflammatory properties [8]. Recently, melatonin was found to exert anti-fibrotic effects in many tissues, including heart, lung, and kidney [11]. Melatonin alleviated myocardial fibrosis in animal models of myocardial infarction [24], pulmonary hypertension [25], and chronic intermittent hypoxia [26]. Bleomycin-induced experimental lung fibrosis was also attenuated by treatment with melatonin [27]. In addition, melatonin has been shown to attenuate kidney fibrosis induced by CCl4 [28], unilateral ureteral obstruction [29], or diabetes [30]. Interestingly, it was also reported that melatonin ameliorated liver fibrosis induced by CCl4 [12,13,14], thioacetamide [15], or high-fat diet [16]. The hepatic fibrotic processes in a bile duct ligation-induced model [17] and a genetic model of primary sclerosing cholangitis [18] were also attenuated by melatonin. However, cellular and molecular mechanisms for the anti-fibrotic effect of melatonin on liver fibrosis remain incompletely understood. In the present study, we demonstrated that melatonin effectively inhibits EMT stimulated by TGF-β1 in AML12 hepatocytes. During fibrotic processes, myofibroblasts synthesize and secret ECM proteins into the liver parenchyma. Hepatic stellate cells and portal fibroblasts have been identified as the main source of hepatic myofibroblasts [1]. In addition, it has been suggested that hepatocytes can also transdifferentiate into myofibroblasts via the EMT process and contribute to the development of liver fibrosis [2,3,4,5]. Thus, our findings provide an important novel mechanism for the preventive action of the hormone on liver fibrosis.

Smad signaling, the canonical TGF-β1 signaling pathway, modulates gene expression required for fibrotic processes including EMT [20]. Interaction of TGF-β1 with its receptor induces Smad2/3 phosphorylation and the phosphorylated proteins assemble into a complex with Smad4. Then, the Smad complex transports to the nucleus in order to modulate the transcriptional expression of fibrosis-associated genes. In the present study, we found that melatonin dose-dependently inhibited Smad2/3 phosphorylation induced by TGF-β1. Besides the canonical Smad cascade, TGF-β1 can activate non-Smad signaling cascades [21]. We found that TGF-β1-stimulated activation of ERK1/2 and p38 was also dose-dependently inhibited by melatonin, whereas JNK1/2 phosphorylation was not affected. Previous studies have shown that Smad and MAPK signaling pathways play an important role in EMT stimulated by TGF-β1 [31,32,33] and the development of liver fibrosis [34,35,36,37]. Taken together, these results suggest that melatonin prevents EMT stimulated by TGF-β1 in AML12 hepatocytes through suppression of Smad and MAPK signaling cascades.

Melatonin exerts various hormonal effects through both membrane receptor-dependent and independent mechanisms [8]. In this study, we used luzindole, a known antagonist of melatonin receptors, to investigate whether melatonin receptors are required for the inhibitory action of the hormone on EMT stimulated by TGF-β1. We found that luzindole failed to block the effects of melatonin, suggesting that melatonin receptors are not required for the suppressive action of the hormone. It has been known that membrane receptor-independent actions of melatonin are associated with its ROS scavenging ability [8]. Because melatonin is highly lipophilic, it can easily pass through the plasma membrane. In the cytosol, it can act as a direct scavenger of ROS. In addition, previous studies revealed the role of ROS in EMT processes of hepatocytes [38,39,40]. In the present study, we also showed that melatonin suppressed ROS generation induced by TGF-β1 treatment and NAC, a well-known antioxidant, also effectively prevented TGF-β1-induced EMT. However, NAC was less potent than melatonin. Although further studies will be required to clarify more detailed molecular mechanisms, our findings suggest that melatonin receptors are dispensable for the inhibitory action of melatonin on EMT stimulated by TGF-β1 and the preventive effect of melatonin is linked to its ROS scavenging property.

## 5. Conclusions

In conclusion, these results suggest that melatonin prevents EMT stimulated by TGF-β1 through suppression of Smad and MAPK signaling pathways by deactivating ROS-dependent mechanisms in a membrane receptor-independent manner. Our data support the notion that melatonin might be a potential preventive agent against liver fibrosis.

## Figures and Tables

**Table 1:** List of primers used in this study.

Gene	Primer Sequence (5′→3′)	Product Size (bp)
E-cadherin	Forward: GACAGAAACGAGACTGGGTCA Reverse: CCGGTGATGCTGTAGAAAACC	130
α-SMA^1^	Forward: GTCCCAGACATCAGGGAGTAA Reverse: TCGGATACTTCAGCGTCAGGA	102
Vimentin	Forward: GATCGATGTGGACGTTTCCAA Reverse: GTTGGCAGCCTCAGAGAGGT	145
Fibronectin	Forward: CGAGGTGACAGAGACCACAA Reverse: CTGGAGTCAAGCCAGACACA	149
GAPDH^2^	Forward: ACTCCACTCACGGCAAATTC Reverse: TCTCCATGGTGGTGAAGACA	171

**^1^** α-smooth muscle actin; **^2^** glyceraldehyce 3-phosphate dehydrogenase.
